# An Uncharted Path of Metastasis: A Case Report of Sigmoid Colon Cancer with Synchronous Vaginal and Urethral Spread

**DOI:** 10.3390/diseases13080251

**Published:** 2025-08-08

**Authors:** John Fernando Montenegro, Giovanna Patricia Rivas Tafur, Miguel Diaz, Diego Fernando Alzate, María Camila Faria, Daniel Florez, Richard Andrés Acuña, Cesar Eduardo, Yamil Liscano

**Affiliations:** 1 Grupo de Investigación en Genética, Fisiología y Metabolismo (GEFIME), Ciencias de la Salud Universidad Santiago de Cali, Cali 760035, Colombia; giovanna.rivas@gclinicadeoccidente.com; 2 Programa de Especialización en Medicina Interna, Facultad de Salud, Universidad Santiago de Cali, Cali 5183000, Colombia; 3 Departamento de Investigación y Educación, Clínica de Occidente S.A., Cali 760046, Colombia; 4 Departamento de Salud, Facultad de Medicina, Universidad Santiago de Cali, Cali 760035, Colombia; miguel1diaz996@gmail.com (M.D.); dfalzate60@gmail.com (D.F.A.); camilafaria-15@hotmail.com (M.C.F.); jkl1999@hotmail.com (D.F.); richard.acuna1999@gmail.com (R.A.A.); cesareduardobermudezm@gmail.com (C.E.); 5 Grupo de Investigación en Salud Integral (GISI), Departamento Facultad de Salud, Universidad Santiago de Cali, Cali 760035, Colombia

**Keywords:** colorectal adenocarcinoma, sigmoid colon cancer, gynecological metastases, panitumumab, palliative chemotherapy

## Abstract

**Background and Objective:** Colorectal cancer (CRC) most commonly metastasizes to the liver and lungs; however, synchronous metastases to pelvic structures such as the vagina and urethra are extremely rare, posing a significant diagnostic and therapeutic challenge. This report describes an unusual case of sigmoid colon adenocarcinoma with synchronous metastases to the vagina and urethra, highlighting its diagnostic evaluation and the value of a multidisciplinary approach. **Methods:** A 59-year-old woman with a history of deep vein thrombosis treated with apixaban presented with chronic constipation and pelvic bleeding. A gynecological evaluation revealed a vaginal lesion. A colonoscopy, biopsy, pelvic magnetic resonance imaging, and molecular profiling were performed. Treatment included chemotherapy (capecitabine and oxaliplatin), panitumumab, and pelvic radiotherapy. **Results:** The biopsy confirmed a moderately differentiated invasive adenocarcinoma in the sigmoid colon with synchronous metastases to the vagina and urethra. Molecular profiling identified a *rat sarcoma virus oncogene* and *BRAF (B-Raf proto-oncogene*), allowing for the use of targeted therapy. The patient achieved a complete response according to RECIST 1.1 criteria and significant symptomatic improvement, including pain reduction, although dosages were adjusted for thrombocytopenia. She is currently continuing palliative treatment with good tolerance and durable symptomatic improvement. **Conclusions:** This case underscores the need to consider unusual metastatic sites in patients with colorectal cancer presenting with gynecological symptoms. Early diagnosis, based on imaging and histology, alongside molecular characterization, is crucial for effective personalized therapy. Multidisciplinary coordination is key to optimizing clinical outcomes in these rare metastatic presentations.

## 1. Introduction

Colon adenocarcinoma is one of the most common neoplasms worldwide. Colorectal cancer (CRC) is the third most frequent type of cancer and the fourth leading cause of cancer-related death globally [1]. Its incidence continues to rise, particularly in the older adult population, and it accounts for over 9% of all cancer cases. The overall 5-year relative survival for patients with metastatic CRC is approximately 15% [1,2].

Approximately 33% of patients with CRC will develop metastases [2]. In stage I, distant metastasis occurs in only 6.5% of cases [3]. Isolated metastases to uncommon sites are extremely infrequent, with only a few cases documented in the literature, at an incidence of 0.1–0.3% [4]. When colorectal cancer metastasizes to the female genital tract, the ovaries are the most common site, followed by the vagina and endometrium [2].

However, for pelvic organ metastases, as in our case, the prognosis is often less favorable, with limited information available in the literature. Direct contiguous spread to the vagina is the most frequently reported route [5]. To date, few cases of colorectal metastasis to the vagina have been documented, although they may be underreported. The average age of presentation was 60.6 years (range: 43–83 years). In a case review including 37 patients, 5.4% presented with vaginal discharge, 2.7% with vaginal spotting, 10.8% with a vaginal mass, and 2.7% with perianal discomfort. A total of 10.8% of cases had no gynecological or colorectal symptoms, and the mass was detected during a routine gynecological examination [1,3].

The liver and lungs are the most frequent sites of metastasis in colorectal cancer. However, in exceptional cases, metastatic spread to the vagina has been reported, a very uncommon location that lacks a standardized treatment [6]. In this context, if the disease is oligometastatic, local treatment such as surgical resection may be considered [5].

The majority of colorectal cancers are adenocarcinomas (90–95%), and their prognosis in the metastatic stage depends on tumor biology, patient status, and treatment response. Targeted therapies such as cetuximab and panitumumab have shown efficacy in tumors without mutations in *KRAS* or *NRAS*. Our case is notable, as it involves potentially curable oligometastatic disease through local resection and epidermal growth factor receptor (EGFR). The absence of *RAS* mutations allowed for a personalized approach. This approach is supported by current research on molecular profiles and dissemination pathways [7,8].

Although there are no conclusive data in the literature, a comparison of dissemination routes in colorectal metastases to the vagina found 50% occurred via the lymphatic route and 40% via the hematogenous route [8]. Four mechanisms have been proposed: direct local invasion, transcoelomic dissemination, retrograde lymphatic pathway, and hematogenous route. However, evidence is limited, and there is no consensus [4,9]. Our case is extremely rare due to its synchronous spread to the vagina and urethra. Urethral metastases from a colonic origin are unusual, and their mechanism is not well-defined. It has been suggested that tumor migration may occur through venous flow to the ovarian plexus or parametrium, or by direct contiguity [5]. Local recurrences are more frequently observed in the vagina, pelvic nodes, cervix, adnexa, and peritoneum [10].

This case describes a rare synchronous metastatic spread of sigmoid colon cancer to the vagina and urethra, without hepatic or pulmonary involvement. The absence of KRAS, NRAS, and BRAF mutations allowed for the successful use of panitumumab. To our knowledge, this is one of the few documented cases showing complete remission of vaginal and urethral lesions in the context of oligometastatic colorectal cancer. It highlights the importance of maintaining a high clinical suspicion for atypical gynecological symptoms, even without signs of pelvic involvement. It emphasizes the value of a complete physical examination and the need for an individualized approach in the absence of specific guidelines, especially regarding local therapies [7,8]. Publishing this case allows for the recognition of unusual dissemination routes, such as contiguity, retrograde lymphatic pathway, hematogenous, and transcoelomic routes. These poorly documented mechanisms can guide early diagnosis. It provides useful evidence for personalized decisions in complex scenarios [4].

The management of colorectal adenocarcinomas with metastases in unusual sites, such as the vagina and urethra, requires a multidisciplinary and individualized approach. The combination of chemotherapy, radiotherapy, and targeted therapies can improve disease control and quality of life. Recognizing atypical gynecological symptoms and applying timely diagnostic tools is key to avoiding delays [8]. Comprehensive clinical evaluation and molecular profiling allow for optimizing diagnosis and guiding personalized therapies effectively.

## 2. Case Report

**Figure 1:**
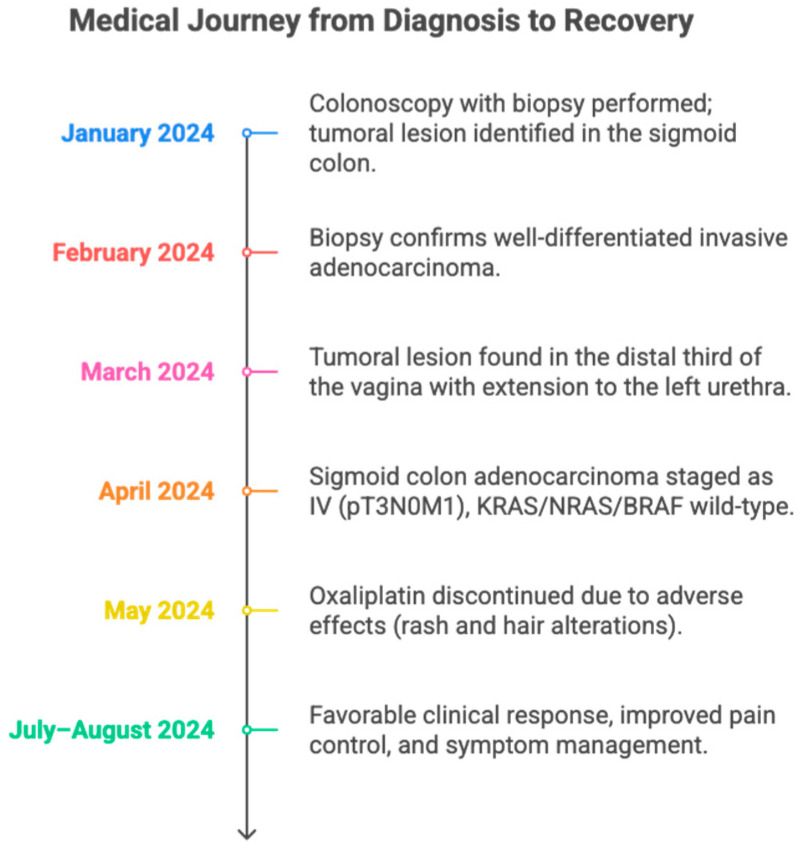
A timeline summarizing the clinical evolution of a patient with sigmoid colon adenocarcinoma metastatic to the vagina and urethra. It details the main diagnostic, therapeutic, and treatment response milestones between January and August 2024. The figure highlights the utility of a multidisciplinary approach and individualized adjustment of oncological management.

The patient is a 59-year-old female with a relevant medical history of deep vein thrombosis, for which she was on anticoagulant therapy with apixaban, and no other significant history (See Figure 1). For approximately 2 years, the patient had been experiencing chronic constipation refractory to conventional laxatives. This symptom was recently joined by the onset of abnormal pelvic and vaginal bleeding, prompting referral to the gynecologic oncology service for specialized evaluation. During the clinical assessment by this service, a physical examination identified a lesion in the vaginal introitus, approximately 2 cm in diameter, a finding that directed the need for additional diagnostic studies.

In January 2024, a colonoscopy with biopsies was performed, revealing a lesion in the middle third of the sigmoid colon, located 3 cm from the nearest margin, 5.5 cm from the farthest margin, and 13 cm from the radial margin. No pre-existing polypoid lesions, lymphovascular compromise, or perineural infiltration were observed.

**Figure 2:**
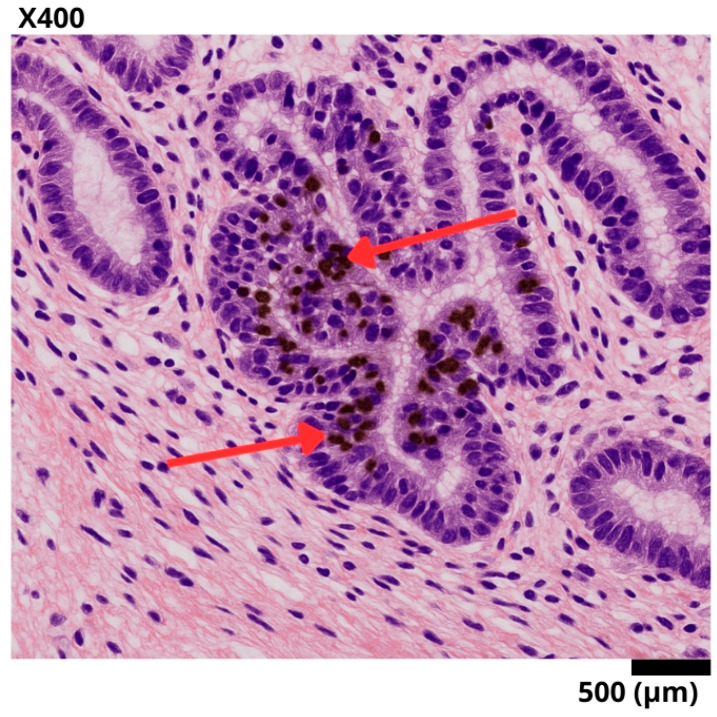
Representative histological section of a sigmoid colon biopsy, stained with hematoxylin–eosin and immunohistochemistry for Ki-67. A well-differentiated invasive adenocarcinoma is observed, with complex glandular architecture and stromal invasion. The red arrows point to tumor nuclei with intense nuclear staining for Ki-67, reflecting a high proliferation index (95%) and suggesting aggressive biological behavior.

In February 2024, the biopsy confirmed a moderately differentiated adenocarcinoma, grade 2. Immunohistochemistry indicated an infiltrating adenocarcinoma with a *Ki-67* proliferation index of 95%. No microsatellite instability was detected (see Table 1) (See Figure 2).

The table presents the results of the colon biopsy, diagnosing a moderately differentiated adenocarcinoma, grade 2. Immunohistochemistry showed negative microsatellite instability, ruling out alterations in DNA deoxyribonucleic acid repair mechanisms.

In March 2024, given the previous findings, staging studies were requested. Abdominal magnetic resonance imaging (MRI) showed no evidence of metastases in the upper abdomen; as an incidental finding, a duplicated right renal collecting system was reported. A pelvic MRI showed concentric mural thickening of the sigmoid colon walls causing partial stenosis, with features suggestive of neoplasia. Additionally, signs of extramural involvement were identified: a tumor lesion in the distal third of the vagina with extension toward the left side of the urethra, and a perilesional lymph node suspicious for metastatic involvement.

The exceptional nature of this case lies in the presence of synchronous metastases to gynecological structures, specifically the vagina and urethra, documented at the time of initial diagnosis. This finding was supported by imaging studies such as pelvic MRI, which revealed tumor involvement of the distal third of the vagina with extension toward the left side of the urethra, without evidence of other distant visceral metastases. Direct or lymphatic spread to external genital structures from a sigmoid colon adenocarcinoma is extremely infrequent and sparsely reported in the literature, which highlights the importance of this case as a relevant clinical contribution. This initial presentation, along with the histological finding of well-differentiated adenocarcinoma and a molecular profile without mutations in *KRAS*, *NRAS*, and *BRAF*, and programmed death-ligand 1 (PD-L1) expression < 1% (CPS < 1) with a low TMB tumor mutational burden (5 mut/Mb), suggested a poor response to immunotherapy, favoring the use of anti-EGFR over checkpoint inhibitors. This allowed for a multidisciplinary and targeted therapeutic approach, which included chemotherapy, biological therapy with panitumumab, and pelvic radiotherapy, with a favorable clinical response.

In April 2024, the patient was evaluated by the oncology service, classifying the case as a stage IV (T3N0M1) sigmoid colon adenocarcinoma with involvement of vaginal and urethral structures. The patient was considered to have an ECOG performance status of 1 and a Karnofsky score of 80%. Molecular studies showed no microsatellite instability or mutations in the *KRAS* or *NRAS* genes, ruling out an indication for immunotherapy. It was decided to start treatment with chemotherapy according to the XELOX protocol (oxaliplatin and capecitabine), with oral capecitabine at a dose of 1000 mg/m^2^ twice daily for 14 days, followed by a 7-day rest period (every 21 days), and oxaliplatin at a dose of 180 mg/m^2^ intravenously on day 1 of each cycle, combined with panitumumab at a dose of 6 mg/kg intravenously every 14 days and radiotherapy at a dose of five fractions of 3 Gy each, for a total of 15 Gy. The patient was also referred to the urologic oncology service for a multidisciplinary approach.

The patient continued specific oncologic therapy with capecitabine and oxaliplatin-based chemotherapy, completing four cycles at the previously mentioned doses. During the administration of the fourth cycle, oxaliplatin was discontinued due to skin reactions and pilar changes, interpreted as possible clinical markers of a good treatment response. These manifestations were managed with topical emollients, without the need to modify other components of the therapeutic regimen.

In June, she began palliative systemic treatment with adequate tolerance and overall clinical improvement. However, she developed asthenia and thrombocytopenia attributable to oxaliplatin, which prompted a progressive dose reduction: initially by 5% to a dose of 170 mg, followed by an additional 15% adjustment to a dose of 145 mg, subsequently finishing treatment at this dose. Given the absence of mutations in the *KRAS* and *NRAS* genes, she was considered eligible for targeted therapy, and combined treatment with panitumumab was initiated. To date, she has received nine cycles of palliative chemotherapy with capecitabine and panitumumab, without major complications and with good clinical tolerance and response.

The RECIST 1.1 criteria were applied. The primary lesion in the sigmoid colon with a baseline measurement of 45 mm showed complete disappearance at the sixth-cycle re-evaluation. The vaginal lesion, initially 25 mm, and the left urethral lesion of 18 mm also disappeared completely in control studies. No new lesions or progression in other areas were identified. According to RECIST 1.1 criteria, these findings are consistent with a complete response (CR).

A biopsy of the vaginal lesions was not performed due to the strong correlation between clinical and radiological findings and therapeutic response. Pelvic MRI revealed lesions consistent with metastases from the sigmoid colon, without hepatic or pulmonary involvement. These lesions resolved following systemic treatment, in parallel with the primary tumor. This supports their metastatic nature. The decision also avoided an invasive procedure in a patient on anticoagulation and with thrombocytopenia.

This case describes a sigmoid colon adenocarcinoma with synchronous metastases to the vagina and urethra, an extremely rare presentation. Metastases to female genitals from colorectal tumors are infrequent and usually related to advanced disease. Abnormal genital bleeding led to the diagnosis through imaging studies and biopsy. This finding highlights the need to suspect metastases in unusual organs. Its rarity justifies its reporting as a relevant clinical contribution.

The absence of specific guidelines for colorectal metastases to the vagina and urethra poses therapeutic challenges, requiring an individualized and multidisciplinary approach. The molecular profile without mutations in *KRAS*, *NRAS*, or *BRAF* allowed the use of panitumumab with a good clinical response. The unusual location of the metastases and the favorable evolution under targeted therapy justify the report. This case contributes to the knowledge of atypical presentations of advanced colorectal cancer.

Currently, the patient continues on palliative systemic treatment, with adequate tolerance and a favorable clinical evolution. She has shown a complete intra-abdominal response, evidenced by imaging studies and correlated with a significant symptomatic improvement. Among the observed adverse effects, acneiform rash-type skin reactions and EGFR-related skin toxicity have been interpreted as positive clinical markers of response to treatment with panitumumab, consistent with what is described in the literature on anti-epidermal growth factor targeted therapies [11].

Pain has been effectively controlled through analgesic adjustment, and the patient’s quality of life has progressively improved. She is currently in the maintenance phase with panitumumab, after the discontinuation of capecitabine, which has allowed the continuation of oncological management with minimal toxicity and adequate adherence to treatment. This clinical course supports the importance of adapting the therapeutic regimen according to the patient’s evolution and reinforces the role of comprehensive monitoring in contexts of metastatic disease with atypical presentations.

## 3. Discussion

### 3.1. Diagnostic Challenges and Clinical Features of an Unusual Colorectal Metastasis

This case highlights the complexity of metastatic colon cancer when it affects unusual organs, which represents a significant diagnostic challenge. Although metastases to atypical sites are infrequent, they can manifest with a wide range of non-specific symptoms depending on the location, making their timely recognition difficult [2]. Unlike the classic pattern of hepatic or pulmonary dissemination, in our patient, the initial symptom was vaginal bleeding, which initially pointed toward a gynecological etiology. Only after detailed imaging studies and histopathological confirmation was the colorectal origin of the lesions established, correlated with a history of vaginal bleeding and imaging findings suggestive of pelvic metastatic lesions [11]. These images raised suspicion of metastasis when considering the patient’s oncological context, reinforcing the need to maintain a high index of suspicion for atypical clinical presentations [12].

### 3.2. Treatment for Unusual Cases

The ASCO 2023 guideline recommends doublet chemotherapy (FOLFOX or FOLFIRI) along with bevacizumab or panitumumab/cetuximab in *RAS* wild-type tumors (without mutations in *KRAS* or *NRAS*) [2]. Gündoğan et al. (2018) described a case of colon adenocarcinoma with vaginal metastasis, treated with laparoscopic hemicolectomy and FOLFOX chemotherapy [1]. Korkmaz et al. (2021) reported four patients with vaginal metastasis secondary to solid tumors, treated with neoadjuvant chemoradiotherapy and 12 cycles of FOLFOX, without reporting microsatellite instability [10]. The ESMO guidelines suggest doublet chemotherapy (FOLFOX, FOLFIRI, or CAPOX) combined with anti-VEGF or anti-EGFR according to the molecular profile [12]. However, more experience from case series is needed on the use of these drugs for metastases to the vagina and urethra, as well as guidelines for metastases in unusual sites like the vagina [10,13].

The absence of mutations enabled effective targeted therapy, reinforcing the value of molecular profiling in personalized treatment, which allowed the use of anti-EGFR targeted therapy (panitumumab). Anti-EGFR therapy is indicated in tumors without microsatellite instability (microsatellite stable, MSS) and with wild-type *RAS*/*BRAF*. In contrast, tumors with high microsatellite instability (microsatellite instability-high) are candidates for immunotherapy.

### 3.3. Unique Aspects of the Case

This case is exceptional due to the synchronous location of metastases in the vagina and urethra, sites rarely affected in colorectal cancer, with scarce cases reported [5]. It presents a molecular profile with MSS and *RAS*/*BRAF* wild-type, which allowed the use of panitumumab as a targeted therapy. Although guidelines do not contemplate this scenario, it has been shown that anti-*BRAF*/EGFR combinations can prolong survival up to 9.3 months. Molecular advances in metastatic CRC allow for specific therapies according to the patient’s genetic subgroup [14]. Vaginal metastases have a poor prognosis, but the patient showed relevant clinical improvement [5]. However, specific guidelines for these cases have not yet been established, as vaginal metastases are considered to have a poor prognosis, although our patient has shown good clinical improvement [5].

Given the atypical pattern of dissemination observed, Batson’s venous plexus is proposed as a plausible route. This valveless network allows retrograde tumor cell spread to pelvic structures, even in the absence of hepatic or pulmonary metastases. This mechanism has been described as an alternative pathway for tumor dissemination in colorectal cancers with gynecological involvement, particularly in oligometastatic disease. Recent studies support its anatomical relevance in the spread of malignancies to the pelvis and perineal region, including the vagina and urethra [15,16]. This system facilitates dissemination from the colon to pelvic structures such as the vagina and urethra, bypassing the hepatic and pulmonary filter. It could explain the synchronous metastasis in the absence of detectable visceral disease. Furthermore, shared mutations in *TP53* tumor protein 53 and *APC* were identified between the primary tumor and metastases, suggesting a common clonal origin. The high intratumoral heterogeneity and multiclonal seeding support simultaneous dissemination routes to multiple sites [17,18].

### 3.4. Review of Similar Cases in the Literature

Several cases have been reported in the medical literature that highlight the diagnostic challenges associated with metastases in uncommon sites. However, reports from Latin America are scarce, which underscores the need for greater documentation and dissemination of these cases. Previous reports are consistent with this uncommon dissemination pattern. For example, Sakhri et al. (2024) describe a 70-year-old patient with vaginal metastasis secondary to rectal adenocarcinoma, who required radical surgical management after local progression [5].

Kwon et al. (2020) report a case of isolated vaginal metastasis in a patient with stage I colorectal cancer, who developed vaginal bleeding 10 months after surgery. The biopsy confirmed colorectal origin, and transvaginal excision was performed without adjuvant treatment. After a local recurrence at 33 months, another surgery was performed. At 54 months, there was no progression, highlighting the importance of considering gynecological metastases even in early stages in the face of persistent symptoms [3].

Urethral metastases from colorectal adenocarcinoma are very infrequent. Karakose et al. (2013) describe the case of a 67-year-old patient who, after surgery and adjuvant treatment, developed urethral metastasis confirmed by histopathology, followed by hepatic involvement. Hematogenous, lymphatic, or urinary dissemination is proposed. Although local resection can alleviate symptoms, its impact on survival is uncertain, requiring a multidisciplinary approach [19]. We present some clinical cases in Table 2.

The findings highlight the importance of considering atypical metastases in patients with a history of colorectal cancer, even with extraintestinal symptoms. Treatment must be individualized and guided by clinical, imaging, and histopathological evaluation [20]. A multidisciplinary approach is essential to optimize management, avoid unnecessary interventions, and prioritize quality of life in palliative contexts.

**Table 2:** Comparison of Cases and Outcomes.

Author	Gende and Age	History	Clinical Manifestation	Procedure Performed	Treatment	Clinical Follow-Up
Saida Sakhri et al., (2024) [5]	Female, 70 years old	Hysterectomy with oophorectomy due to fibroid	Perineal discomfort, abdominal fullness, mild rectal bleeding	Anterior resection with total mesorectal excision + ileostomy	Adjuvant chemotherapy	Clinically stable
Soon Keun Kwon et al., (2020) [3]	Female, 63 years old	Colorectal cancer	Vaginal spotting	Vaginal excision and abdominal MRI	Surgical treatment	Recurrence-free
Gündoğan et al., (2018) [1]	Female, 59 years old	Arterial hypertension	Vaginal discharge 1 year after hemicolectomy	Left hemicolectomy	FOLFOX6 (chemotherapy) + ileostomy closure at 6 months	Favorable follow-up
Efetov et al., (2024) [21]	Female, 61 years old	No significant medical history	Pelvic pain and purulent genital discharge, mass detected in fallopian tube and uterus	Total hysterectomy with bilateral salpingo-oophorectomy	Adjuvant chemotherapy CAPOX (5 cycles at time of report)	Partial response

MRI: Magnetic Resonance Imaging; FOLFOX6: Folinic acid (leucovorin), Fluorouracil (5-FU), and Oxaliplatin (6-cycle regimen); CAPOX: Capecitabine and Oxaliplatin; TME: Total Mesorectal Excision.

In cases of colorectal adenocarcinoma with synchronous metastasis to the vagina and/or urethra, there are no large series to establish a clear median survival; however, data extracted from case reports and systematic reviews suggest that overall survival (OS) can vary from 8 to 20 months depending on the number of metastases, the molecular profile, and the type of treatment received. Progression-free survival (PFS), when targeted therapies are used in *RAS*/*BRAF* wild-type tumors (such as panitumumab), can reach 5 to 10 months in oligometastatic disease with good functional performance (ECOG 0–1) [17,22].

The case’s molecular profile—*KRAS*/*NRAS*/*BRAF* wild-type, MSS, high *Ki-67*—had a good response to chemotherapy, complete response according to RECIST 1.1, no liver or lung metastases, and good tolerance to anti-EGFR, supporting a favorable prognosis. These findings are consistent with previous descriptions and reinforce the utility of an individualized therapeutic approach [18].

### 3.5. Therapeutic Considerations

The choice of XELOX over FOLFOX in our case is justified by its equivalent efficacy, reduced need for hospitalization, and better outpatient tolerance, by avoiding continuous 5-FU infusion and the use of central venous access [23]. Recent studies show that XELOX offers overall and progression-free survival comparable to FOLFOX, with manageable toxicity profiles. In patients with good tolerance and response to targeted therapy, as in this case, it represents a cost-effective alternative. In oligometastatic colorectal disease, surgery plays an important role, as it can offer curative benefit or prolong survival when there is a good systemic response and adequate functional status [24]. In the absence of liver or lung metastases, as in our case, pelvic surgery can be considered after a partial or complete response [25]. In this same context, pelvic radiotherapy or brachytherapy can be useful for local control, especially if surgery is not viable, alleviating symptoms such as pain or bleeding [6]. Furthermore, with a favorable molecular profile (*RAS*/*BRAF* wild-type), radiotherapy can consolidate the local response and delay progression, always as part of a multidisciplinary approach [2].

### 3.6. Multidisciplinary Management Approach

The integration of imaging studies, immunohistochemistry, and targeted biopsies confirmed the presence of metastatic adenocarcinoma with involvement of unusual pelvic structures, including gynecological organs, which, although infrequent, is documented in the literature [2]. In patients with a history of adenocarcinoma, especially those who are immunocompromised, it is essential to maintain a high index of suspicion for non-specific pelvic symptoms, as they could correspond to metastases in atypical sites. This early recognition is key to avoiding diagnostic delays and incorrect clinical decisions that could negatively affect the patient’s prognosis [12]. These decisions should be guided by specific clinical findings that direct personalized treatment, improving the therapeutic response and, potentially, survival [9,21].

Oncological treatment was progressively adjusted based on clinical findings, implementing personalized chemotherapy and targeted therapy regimens according to the clinical and immunological profile of the case [8]. This individualized strategy not only contributed to a better therapeutic response but also helped preserve quality of life, which is especially relevant in contexts where the focus is palliative [9].

In the multidisciplinary approach to unusual pelvic metastases, it is essential to consider the possible routes of tumor dissemination [22]. Although colorectal cancer usually metastasizes via the hematogenous route to the liver and lungs, mechanisms such as retrograde lymphatic dissemination or direct extension from rectosigmoid masses could also explain the involvement of pelviperineal structures. Even though not evidenced in this case, tumor seeding secondary to surgical or endoscopic interventions should be considered. Recognizing these dissemination pathways allows for a better interpretation of clinical findings and guides more precise surgical and therapeutic decisions within the treating team [22,23].

Molecular analysis identified a *KRAS*/*NRAS* wild-type tumor, which allowed the use of panitumumab as a targeted therapy. This decision was based on its efficacy in tumors without mutations in those pathways. Furthermore, the presence of atypical pelvic metastases in the vagina and urethra led to a rethinking of the diagnostic approach. This highlighted the need for a molecular profile in metastatic disease and for multidisciplinary integration. Thus, a more precise and personalized care is favored [2].

In the multidisciplinary management approach, it is recommended to start with a structured diagnostic algorithm that includes gynecological evaluation, colonoscopy with biopsy, pelvic MRI, and complete molecular profiling (*KRAS*, *NRAS*, *BRAF*, MSI/MMR), which allows for confirming the colorectal origin of atypical metastases and guiding treatment accurately [25]. Subsequently, rigorous clinical and imaging follow-up should be established every 8 to 12 weeks, using tools such as MRI or positron emission tomography-computed tomography (PET-CT), along with tumor markers and continuous evaluation by gynecologic oncology. In patients with oligometastatic disease confined to the vagina or urethra, without liver or lung involvement, with a good response to systemic treatment (partial or complete response by RECIST), and an Eastern Cooperative Oncology Group (ECOG) functional status of 0–1, the use of local therapies such as surgery or radiotherapy should be considered, aiming for locorregional control or even a curative objective [17,24].

### 3.7. Limitations and Future Research

The main limitation of this type of case lies in its low frequency, which can lead to a diagnostic underestimation in clinical practice. Current evidence is primarily based on case reports, which limits the generalizability of the findings. Nevertheless, these reports highlight the need to consider unusual pelvic metastases, such as vaginal or urethral, even in early stages of colorectal cancer. A biopsy of the vaginal lesions was not performed due to strong clinical and radiological correlation and complete therapeutic response. This decision also considered the patient’s anticoagulation and thrombocytopenia.

Future multicenter and collaborative research is necessary to better characterize the clinical behavior, dissemination mechanisms, and therapeutic responses, promoting more effective diagnostic and therapeutic strategies in complex clinical scenarios. The rarity of these metastases limits available clinical evidence, making multicenter studies essential to establish specific diagnostic and treatment guidelines [2].

## 4. Conclusions

This case report presents an unusual pattern of metastasis from sigmoid colon adenocarcinoma to infrequent pelvic structures, such as the vagina and the urethra. Its atypical presentation underscores the importance of maintaining a high level of diagnostic suspicion in patients with a history of colorectal cancer who present with non-specific gynecological symptoms.

The combined use of imaging studies, biopsy, and immunohistochemistry was crucial for confirming the diagnosis and guiding appropriate treatment. Furthermore, the molecular characterization of the tumor as *RAS*/*BRAF* wild-type enabled the use of targeted therapies, improving the precision of the therapeutic approach.

## Figures and Tables

**Table 1:** Biopsy Results.

Examination	Results
Colon biopsy	Moderately differentiated adenocarcinoma, grade 2
Microsatellite Instability Immunohistochemistry	The mutational profile showed wild-type *KRAS*, *NRAS*, and *BRAF*, with a mutation in *TP53* (p.R175H) and negative *PIK3CA*. *PD-L1* expression was <1% (CPS < 1), and the TMB was low (5 mut/Mb)

## Data Availability

Data are contained within the article.

## Abbreviations

CCR	Colorectal Cancer
EGFR	Epidermal Growth Factor Receptor
ADN	DNA (Deoxyribonucleic Acid)
RMN	MRI (Magnetic Resonance Imaging)
CEA	Carcinoembryonic Antigen
XELOX	Chemotherapy regimen with capecitabine and oxaliplatin
CAPOX	Alternative name for the XELOX regimen
FOLFOX	Chemotherapy with folinic acid, fluorouracil, and oxaliplatin
FOLFIRI	Chemotherapy with folinic acid, fluorouracil, and irinotecan
RAS wild-type	State without mutations in KRAS or NRAS genes
Ki-67	Cell proliferation index (immunohistochemical marker)
KRAS/NRAS	Genes related to resistance to anti-EGFR targeted therapy
IDH	Histological Density Index
TME	Total Mesorectal Excision
PFS	Progression-Free Survival
